# Assessment of water contamination by potentially toxic elements in mangrove lagoons of the Red Sea, Saudi Arabia

**DOI:** 10.1007/s10653-021-00956-5

**Published:** 2021-05-26

**Authors:** Dhafer Ali Alamri, Samir G. Al-Solaimani, Refaat A. Abohassan, Jörg Rinklebe, Sabry M. Shaheen

**Affiliations:** 1 Department of Arid Land Agriculture, Faculty of Meteorology, Environment, and Arid Land Agriculture, King Abdulaziz University, Jeddah, 21589 Saudi Arabia; 2 Laboratory of Soil and Groundwater-Management, School of Architecture and Civil Engineering, Institute of Foundation Engineering, Water and Waste Management, University of Wuppertal, Pauluskirchstraße 7, 42285 Wuppertal, Germany; 3 Department of Environment, Energy and Geoinformatics, Sejong University, Seoul, 05006 Republic of Korea; 4 Department of Soil and Water Sciences, Faculty of Agriculture, University of Kafrelsheikh, Kafr El-Sheikh, 33516 Egypt

**Keywords:** Water pollution, Heavy metals, Mangrove forests, Risk assessment, Human and environmental health

## Abstract

Mangrove (*Avicennia marina*) forests in the Red Sea cost have great concern from environmental, biological, economic, and social points of view. Therefore, assessing water contamination in this ecosystem is worth to be investigated. Consequently, here we aimed to examine the levels of salinity, acidity, and the total content of Fe, Mn, Cu, Zn, Cd, Cr, Ni, and Pb in water samples collected from the upper, middle, and lower part of three mangrove lagoons (i.e., Al-Shuaiba, Yanbu, and Jeddah), Red Sea, Saudi Arabia. The total metal content (µg L^−1^) in water samples differed significantly among the studied areas and ranged from 286.2 to 4815.0 for Fe, 86.4–483.0 for Mn, 22.9–468.8 for Cu, 199.2–366.6 for Zn, 44.1–99.8 for Cd, 25.6–80.3 for Cr, 11.6–41.5 for Ni, and from 17.7 to 102.0 for Pb. The mean values of Cu, Zn, Cd, and Pb were higher than the WHO water quality standards for fisheries. Water samples in Yanbu were more contaminated and contained higher concentrations of all metals than Jeddah and Al-Shuaiba, due to the petrochemical industries in this industrial area. Our findings suggest that the high metal content in the water of these mangrove sites, particularly in Yanbu, should be considered due to the high potential environmental and human health risks in these ecosystems. These results may help for demonstrating effective approaches for the management of these lagoons. More studies will be carried out on the sediment and mangrove plants in this ecosystem.

## Introduction

Contamination of aquatic environment by potentially toxic elements (PTEs) is a critical concern due to their potential toxicity and accumulation in aquatic habitats. Mangrove (*Avicennia marina*) lagoons have great concern from environmental, biological, economic, and social points of view (Constance et al., 2021; FAO, 2007; Leite et al., 2021; Long et al., 2021). The Mangrove lagoons in the Red Sea cost are productive but stressed by high temperature, high salinity, human activities, and potential pollution; therefore, they need continuous monitoring (Aljahdali & Alhassan, 2020; Alzahrani et al., 2018; Rasul, 2015a, b; Sultan & Ahmad, 1990). The anthropogenic activities and the utilization of the coasts may increase the level of pollutants including PTEs in these ecosystems (Albarakati & Ahmad, 2019; Alzahrani et al., 2018; Imaz-Lamadrid et al., 2019; Martínez-López et al., 2020).

Several studies have been carried out on PTEs pollution in water, sediments, and plants in mangrove environments worldwide (e.g., Bakshi et al., 2019; Bodin et al., 2013; Chi et al., 2019; Fernández-Cadena et al., 2014; He et al., 2014; Li et al., 2016). Specifically, some studies (e.g., El-Said & Youssef, 2013; Usman et al., 2013; Alzahrani et al., 2018; Aljahdali and Alhassan, 2020) have been carried out on the mangrove lagoons in the red sea. However, those studies were mainly focused on the contamination of the sediments. Few studies (e.g., Hamed & Emara, 2006) examined the level of PTEs in water samples; however, they examined the concentration of dissolved PTEs, and they did not study the total metal content in water. Consequently, the total PTE content in the water of mangrove lagoons is worth to be investigated.

Among the mangrove lagoons in the red sea in Saudi Arabia, examining the total PTE content in the water of the lagoon of the industrial city Yanbu and the lagoon of Jeddah (the biggest Red Sea coastal city in Saudi Arabia) is very urgent and critical because the first one (Yanbu) receives big amounts of industrial wastewater and the second one (Jeddah) receives huge amounts of sewage effluents as compared to some other lagoons like Al-Shuaiba. Consequently, here we aimed to examine the levels of salinity, acidity, and the total content of eight potentially toxic elements (Fe, Mn, Cu, Zn, Cd, Cr, Ni, and Pb) in water samples collected from the upper, middle, and lower part of mangrove forest lagoons in Yanbu, Jeddah and Al-Shuaiba in the Red Sea coast, Saudi Arabia.

## Materials and methods

### Studied areas

**Fig. 1:**
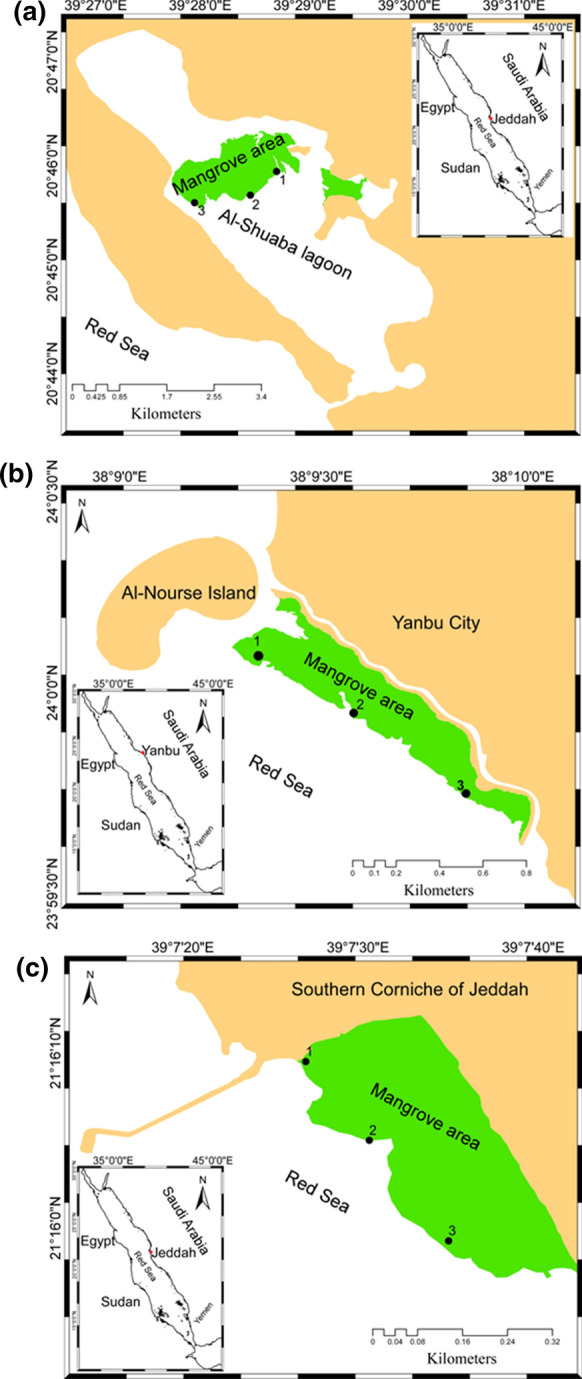
Maps of the studied locations: **a** Al-Shuaiba, **b** Yanbu, and **c** Jeddah

The coastline of the Kingdom of Saudi Arabia is about 1,840 km in length, accounting for 79% of the eastern seaboard of the Red Sea (Badr et al., 2009; MEPA/IUCN, 1987). There are over twenty elongated and shallow lagoons extending along the Saudi the Red Sea coast (Basaham et al., 2019; Rasul, 2015a, 2015b). In our study, we selected three lagoons, i.e., Jeddah, Yanbu, and Al-Shuaiba. Jeddah is one of the biggest Red Sea coastal Cities in Saudi Arabia. The studied site located at Corniche area and its 22 km south of Jeddah (21.3207° N and 21.3483° N; Fig. 1). More details about this location are included in previous studies (e.g., El Sayed, 2002; Basaham et al., 2009). Al-Shuaiba lagoon (14.3 km^2^; 20° 46′ 2″ N and 39° 30′ 21″ E) is located in the eastern coast of the Red Sea, Saudi Arabia (80 km south of Jeddah city) (Fig. 1). More details about this location are included in previous studies (e.g., Abohassan, 2013; Abu-Zied et al., 2011a, 2011b; Basaham et al., 2019). Yanbu is an industrial city on the eastern coast of Red Sea. The estimated area of Yanbu lagoon (24° 02′ 65″ N and 38° 09′ 46″E) is approximately 7500 km^2^. More details about this location are included in previous studies (e.g., Al-Barakati, 2012; Abohassan, 2013; Alharbi et al,. 2019).

### Sampling, characterization, and metal content

In each site, four transects have been set in the north–south orientation. Three water samples were randomly collected from three different points at the beginning (upper; site 1), middle (site 2), and end (lower; site 3) of transect (Fig. 1). Water samples (about 2L) were collected and tightly sealed in polyethylene bottles and transferred to the laboratory. Water salinity and pH were measured using EC- and pH-meter. A part of the water samples was acidified by nitric acid (HNO_3_) for elemental analyses. Fifty milliliters of each acidified water sample was digested with a mixture of concentrated nitric acid (HNO_3_; 5 ml) and perchloric acid (CHlO_4_; 2 ml) for analyses of the total metal content in the water.

The mixture of water and acids was left overnight and then heated in the next day, and the temperature was gradually increased from 100 to 225 °C over a period of 6 h until full digestion. Distilled water was then added into each tube containing sample solution up to 50 ml and filtered through acid-resistant filter paper, as described in the standard methods for the examination of water and wastewater (APHA, 2005) and used also by Kopp and Korner (1967) and Shaheen and Tsadilas (2009). The total metals were measured using graphite furnace—atomic absorption spectrophotometer (Shimadzu AA7000).

### Quality assurance, statistical analyses, and creating figure

The used glasswares were water and/or acid cleaned, and the used chemicals were analytical reagent grade. The water salinity, acidity, and metal content were analyzed in triplicates using calibrated equipments with acceptable uncertainties. The laboratory usually applies an internal quality assurance system. During metal analyses, different concentration levels (e.g., 1000, 100, 50, 25, and 12.5 µg L^−1^) were measured as an internal quality control. The RSD values were less than 5%. The limits of detection (LOD) obtained for Cu, Fe, Mn, Pb, and Zn were 5.4, 6.2, 1.4, 12.0, and 1.8 µ L^−1^, respectively.

Descriptive statistics were performed using the SPSS 22 software (SPSS, Chicago, IL, USA). All results were analyzed statistically using one-way ANOVA to compare the means of different between the systems. The individual means were compared by Duncan’s test to a level of 5% using SPSS version 22. OriginPro 9.1 b215 (OriginLab Corporation, Northampton, USA) was used to create the figures.

## Results and discussions

### Water salinity and pH

**Fig. 2:**
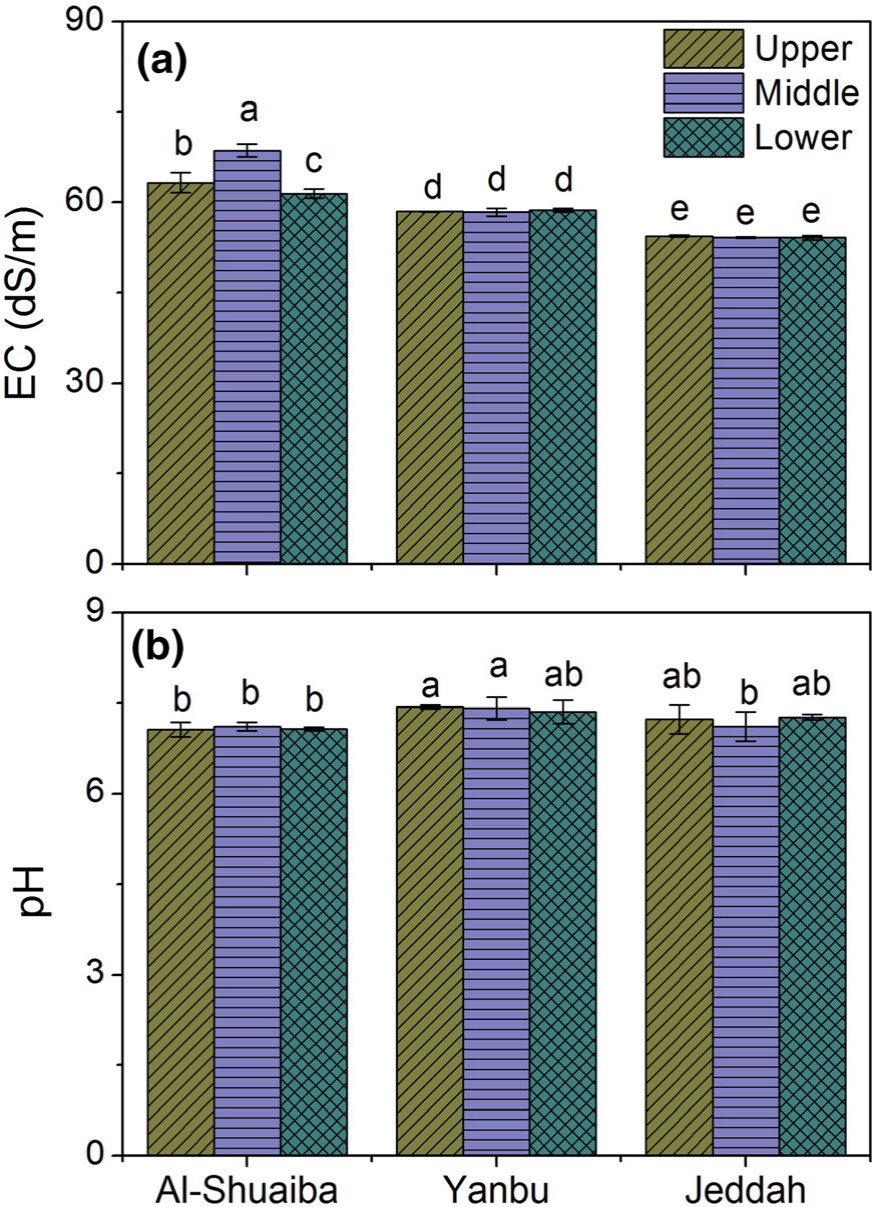
Average values of electrical conductivity and pH in the water samples of the upper, middle, and lower part of the studied lagoons. Values accompanied by different letters are significantly different within columns at the level (*P* < 0.05)

The studied water samples in the three lagoons were salty with electrical conductivity (EC; dS/m) values differed significantly among the sites and varied from 53.8 in Jeddah to 69.4 in Al-Shuaiba (Table 1). The average EC values were 54.2 dS/m, 58.5 dS/m, and 68.2 dS/m in Jeddah, Yanbu, and Al-Shuaiba, respectively (Table 1). Among the different sampling points (upper, middle, and lower) in each site, the water salinity of the middle part of Al-Shuaiba lagoon was significantly higher than the upper and lower part (Fig. 2a), while there were no significant variations between the points in Yanbu and Jeddah lagoons (Fig. 2a). In general, the high water salinity in the three sites of the Red Sea might be explained by the high temperature (tropical–subtropical climate) and high evaporation and low precipitation. Also, there is no dilution for this high saline sea water with Rivers water because the Red Sea is a semi-enclosed, marginal basin and having micro-tidal conditions, and thus, no rivers or streams are connected with the Red Sea and its lower connection to the Gulf of Aden is narrow (Abu-Zied & Bantan, 2013; Morcos, 1970). Among the three lagoons, Al-Shuaiba water showed the highest salinity (Fig. 2). Al-Shuaiba Lagoon is a sheltered, fossil back-reef, hyper-saline water body (Abu-Zied & Bantan, 2013; Abu-Zied et al., 2011a, 2011b). The relatively lower salinity in Jeddah lagoon water can be explained by dilution the saline water with wastewater from the urbanized surrounding area.

**Table 1:** Variations of EC, pH, and metal concentrations in the water samples (*n* = 9) of the studied lagoons

Parameter	Unit	Minimum	Maximum	Mean	Standard deviation
*Al-Shuaiba lagoon*					
EC	dS m^−1^	60.70	69.40	64.43	3.39
pH	–	6.99	7.21	7.09	0.08
Fe	[µg L^−1^]	286.2	822.0	568.8	142.4
Mn		86.4	170.4	115.9	24.2
Cu		22.9	234.9	117.4	55.8
Zn		199.2	241.6	220.8	14.0
Cd		44.1	52.6	46.7	3.1
Cr		45.7	71.5	56.3	9.0
Ni		11.6	20.9	15.2	3.3
Pb		17.7	42.0	31.4	6.9
*Yanbu lagoon*					
EC	dS m^−1^	57.60	59.00	58.50	0.40
pH	–	7.14	7.56	7.41	0.14
Fe	[µg L^−1^]	1321.5	4815.0	2367.2	1199.1
Mn		186.0	483.0	264.8	86.7
Cu		243.8	445.1	376.0	71.7
Zn		331.5	366.6	347.2	13.4
Cd		82.9	99.8	92.2	6.5
Cr		49.6	80.3	68.2	12.2
Ni		19.1	41.5	32.5	6.5
Pb		36.8	102.0	69.9	19.7
*Jeddah Lagoon*					
EC	dS m^−1^	53.80	54.50	54.20	0.23
pH	–	6.84	7.46	7.21	0.18
Fe	[µg L^−1^]	938.6	1872.0	1452.2	285.3
Mn		133.2	222.0	184.4	30.1
Cu		76.5	468.8	240.9	119.0
Zn		241.0	286.0	267.4	12.2
Cd		56.5	85.8	72.0	9.7
Cr		25.6	59.5	40.7	11.6
Ni		18.6	26.7	23.4	2.5
Pb		19.2	70.6	50.9	17.8

*EC* Electrical conductivity

The pH of the studied water samples in the three lagoons varied from 6.84 in Jeddah to 7.56 in Yanbu (Table 1). The average pH values were 7.4, 7.2, and 6.8 in Yanbu, Jeddah, and Al-Shuaiba, respectively (Table 1). Among the different sampling points (upper, middle, and lower) in each site, the water pH of the middle part of Al-Shuaiba lagoon was significantly higher than the upper and lower part (Fig. 2b), while there were no significant variations between the points in Yanbu and Jeddah lagoons (Fig. 2b). The higher pH in Yanbu lagoon water than the other lagoons might be due to the discharge of alkaline industrial wastewater into the lagoons (Alharbi et al., 2019).

### Total metal content in water

**Fig. 3:**
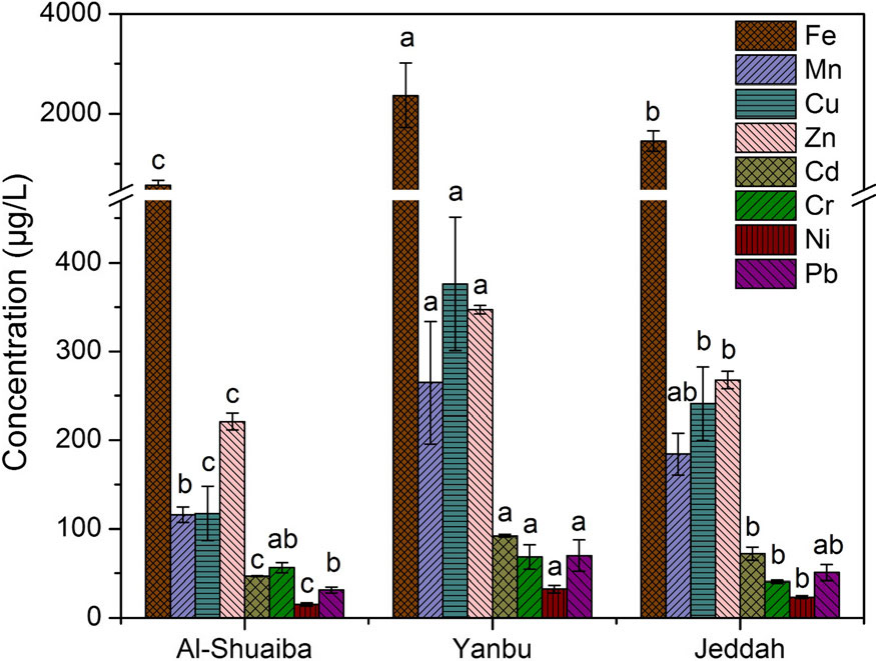
Average values of the total content of Fe, Mn, Cu, Zn, Cd, Cr, Ni, and Pb in the water samples of the studied lagoons. Each metal values accompanied by different letters are significantly different within lagoons at the level (*P* < 0.05)

**Fig. 4:**
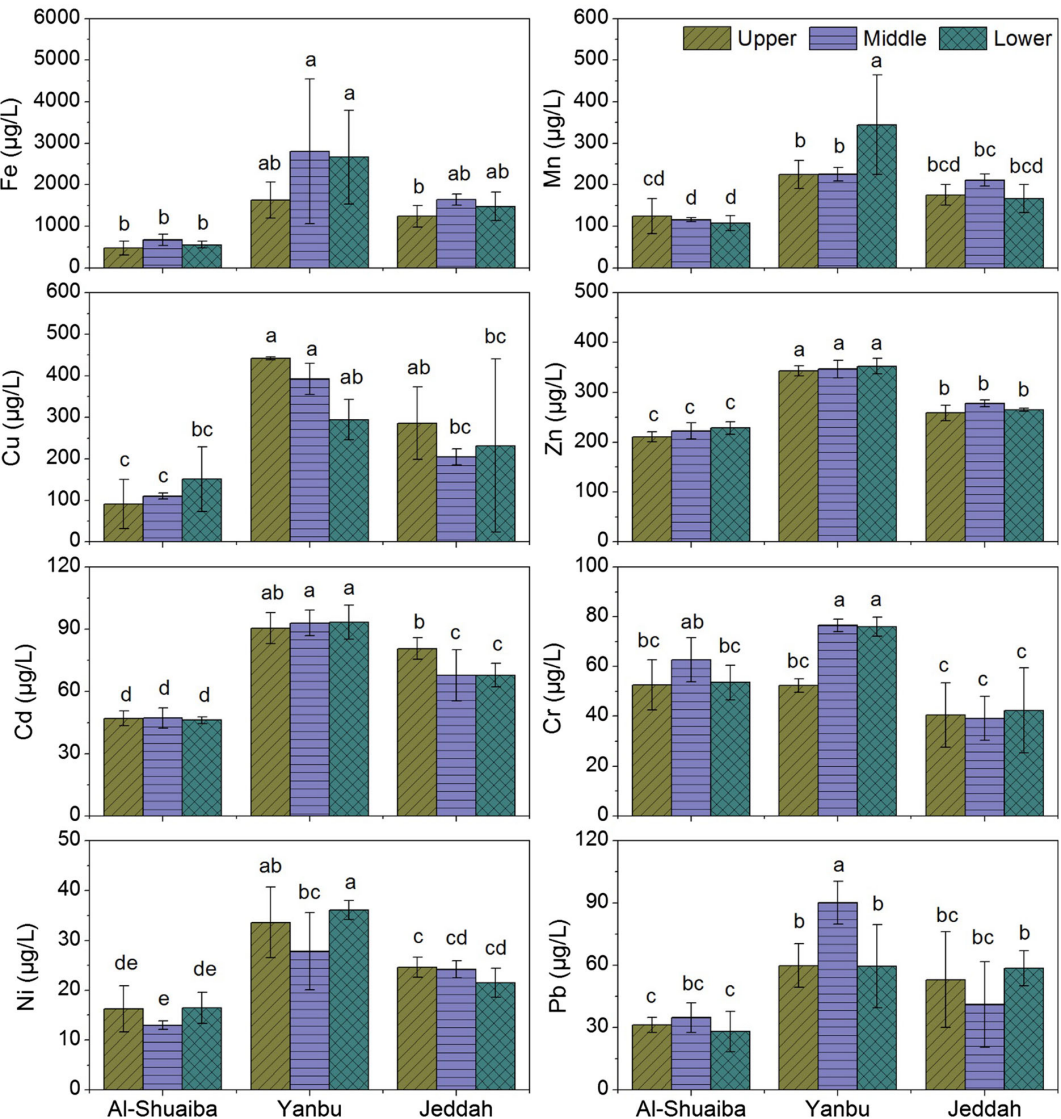
Average values of the total content of Fe, Mn, Cu, Zn, Cd, Cr, Ni, and Pb in the water samples of the upper, middle, and lower part of the studied lagoons of the studied lagoons. Each metal values accompanied by different letters are significantly different within parts and lagoons at the level (*P* < 0.05)

The total metal content in the water samples varied widely among the sites and metals (Fig. 4). Among the studied metals, Fe showed the highest average values in the three lagoons (Fig. 3), followed by Zn, Cu, Mn, Cr, Cd, Pb, and Ni in Al-Shuaiba lagoon, by Zn, Cu, Mn, Cd, Pb, Cr, and Ni in Yanbu Lagoon, and followed by Cu, Zn, Mn, Cd, Pb, Cr, and Ni in Jeddah Lagoon (Fig. 3). The higher Fe content in the water than Zn, Cu, Mn, Cd, Pb, Ni, and Cr can be due to either geogenic or anthropogenic sources. The geogenic inputs might be due to the enrichments of the lagoon sediments by Fe-(oxyhydr)oxides, which can be reductively dissoluted under the flooding conditions, and therefore, Fe as well as the other bounded/occluded meals can be released to the water (Shaheen et al., 2014, 2020). Also, the suspended colloidal particles in the water bodies of the lagoons can be rich in the Fe-(oxyhydr)oxides, which increase the particulate and total content of Fe in the water (Shaheen et al., 2013; Cusack et al., 2020). The enrichment of red sea sediments by total Fe has been reported in some studies (e.g., Basaham et al., 2009; Aljahdali and Alhassan, 2020). The anthropogenic sources for Fe and heavy metals can be due to the discharge of industrial, municipal, and sewage wastewater into the lagoons as reported in other studies (e.g., El Sayed, 2002; Basaham et al., 2009; Alharbi et al., 2019; Cusack et al., 2020) and as it will be discussed below.

The concentrations (µg L^−1^) of total Fe in the water samples differed between the three lagoons and varied from 286.2 in Al-Shuaiba to 4815.0 in Yanbu (Table 1). The variations between the average concentrations of Fe (568.8–2367.2 µg L^−1^) in the three lagoons differed significantly (Fig. 3). Also, the variations between the average concentrations of Fe in the upper, middle, and lower part of each lagoon differed significantly in Yanbu and Jeddah lagoons, but were nonsignificant in Al-Shuaiba lagoon (Fig. 4).

The concentrations (µg L^−1^) of total Mn in the water samples differed between the three lagoons and varied from 86.4 in Al-Shuaiba to 483.3 in Yanbu lagoon (Table 1). The average concentrations of Mn (264.8 µg L^−1^) in Yanbu water were significantly higher than Jeddah and Al-Shuaiba water (Fig. 3). Although the upper part showed significantly higher concentrations of Mn than the middle and lower part of Al-Shuaiba lagoon, the lower part showed significantly higher concentrations than the upper and middle part of Yanbu lagoon, while the middle part showed significantly higher concentrations than the upper and lower part of Jeddah lagoon (Fig. 4).

The total content (µg L^−1^) of total Cu ranged between 22.9 in Al-Shuaiba and 468.8 in Jeddah (Table 1). The average concentrations of Cu (376.0 µg L^−1^) in Yanbu water was significantly higher than Jeddah (240.9 µg L^−1^) and Al-Shuaiba water (117.4; Fig. 3). The upper part was more contaminated by Cu than the middle and lower part of Yanbu and Jeddah lagoons, while the lower part showed significantly higher Cu concentrations than the upper and middle part of Al-Shuaiba lagoon; however, the variations between Cu concentrations in the upper, middle, and lower parts of the lagoons were nonsignificant (Fig. 4).

The total content (µg L^−1^) of Zn ranged between 199.2 in Al-Shuaiba and 366.6 in Yanbu (Table 1). The average concentration of total Zn in Yanbu water (347.2 µg L^−1^) was significantly higher than Jeddah (267.4 µg L^−1^) and Al-Shuaiba water (220.8 µg L^−1^) (Fig. 3). The variations between total Zn content in the lower, upper, and middle parts were nonsignificant in the three lagoons (Fig. 4).

The concentrations of total Cd in the water samples varied from 44.1 µg L^−1^ in Al-Shuaiba lagoon to 99.8 µg L^−1^ in Yanbu lagoon (Table 1). The average concentrations of total Cd in Yanbu water (92.2 µg L^−1^) were significantly higher than Jeddah (72.0 µg L^−1^) and Al-Shuaiba water (46.7 µg L^−1^) (Fig. 3). The variations of total Cd content among the upper, middle, and lower parts of the lagoons differed significantly in Jeddah lagoon, but were nonsignificant in Al-Shuaiba and Yanbu lagoons (Fig. 4).

The total content (µg L^−1^) of total Cr ranged between 25.6 in Jeddah and 80.3 in Yanbu lagoon (Table 1). The average values of total Cr content in Yanbu water (68.2 µg L^−1^) and Al-Shuaiba water (56.3) were significantly higher than Jeddah water (40.7) (Fig. 3). The variation between Cr average concentrations in the upper, middle, and lower part of the lagoons was significant in Yanbu water, while it was nonsignificant Al-Shuaiba and Jeddah lagoons (Fig. 4).

The concentrations (µg L^−1^) of total Ni in the water samples varied from 11.6 in Al-Shuaiba to 41.5 in Yanbu lagoon (Table 1). The average concentrations of total Ni in Yanbu water (32.5 µg L^−1^) and Jeddah water (23.4 µg L^−1^) were significantly higher than Al-Shuaiba water (15.2 µg L^−1^) (Fig. 3). The variation of total Ni content among the upper, middle, and lower parts differed significantly in Yanbu lagoon, while it was nonsignificant in Al-Shuaiba and Jeddah lagoons (Fig. 4).

The total content (µg L^−1^) of Pb in the water samples ranged between 17.7 in Al-Shuaiba lagoon and 102.0 in Yanbu lagoon (Table 1). The mean values of total Pb in were 69.9 µg L^−1^ in Yanbu water, 50.9 µg L^−1^ in Jeddah water, and 31.4 µg L^−1^ in Al-Shuaiba water (Fig. 3). The mean value in Yanbu lagoon water was significantly higher Al-Shuaiba and Jeddah lagoons (Fig. 3). The water of the middle part in Al-Shuaiba and Yanbu lagoons contains higher Pb than the upper and lower part, while the water of the lower part in Jeddah lagoon contains higher Pb than the upper and middle part; however, the variations between total Pb content in the parts were significant only in Yanbu lagoon (Fig. 4).

The metal values were within the global range of metal content in surface water bodies as reviewed and reported by Kumar et al. 2019 (Table 2). The mean values of Fe, Mn, Cu, Zn, Cd, and Ni were lower than the highest permitted value for drinking water according to the world health organization, while the mean values of Cr and Pb was higher than those limits (Table 2) (WHO, 2017). The mean values of Mn, Cd, Ni, and Pb were higher than the highest permitted value for drinking water according to the US Environmental Protection Agency (USEPA), while the mean values of Fe, Cu, Cr, and Zn was lower than those limits (Table 2) (USEPA, 2009). Also, the mean values of Cu, Zn, Cd, and Pb were higher than the water quality standards for fisheries according to the WHO (Wang et al., 2021; WHO, 2011). The high concentrations of these metals in the water samples of the studied lagoons may pose a great threat to these ecosystems.

**Table 2:** Average metal content in the studied lagoons in comparison with the metal content in surface water bodies in different countries worldwide and the pollution level based on WHO and ESPA guidelines for drinking water and fishers

Locations/water quality criteria and standards	Fe	Mn	Cu	Zn	Cd	Cr	Ni	Pb	References
	µg/L								
Al-Shuaiba lagoon	568.8	115.9	117.4	220.8	46.7	56.3	15.2	31.4	This study
Jeddah lagoon	1452.2	184.4	240.9	267.4	72.0	40.7	23.4	50.9	This study
Yanbu lagoon	2367.2	264.8	376.0	347.2	92.2	68.2	32.5	69.9	This study
Metal content in more than 500 surface water bodies in different countries worldwide	0.001–63,500 (1654)*	0.015–77,000 (2562)	0.0007–27,400 (538)	0.01–54,000 (723)	0.003–13,700 (181)	0.001–21,800 (413)	0011–38,100 (946)	–	Kumar et al., 2019
Highest permitted value for drinking water	1000	1000	3000	5000	100	5	70	10	WHO, 2011; Kumar et al., 2019; Wang et al., 2021
Highest permitted value for drinking water	3000	50	1300	5000	5	100	15	15	USEPA 2009; Kumar et al., 2019
Water quality standard for fisheries GB 11607–89	–	–	10	100	5	100	50	50	WHO, 2011; Wang et al., 2021

*Average values

The water samples in Yanbu site were more contaminated and contained higher concentrations of all metals than Jeddah and Al-Shuaiba. The high contamination in the water of Yanbu might be due to the petrochemical industries in this industrial area. Yanbu is an industrial city and contains the industrial harbor, the largest oil transport harbor, oil refineries, petrochemical factories, cement factories, desalination plants, and power generation plants (Al-Barakati, 2012; Abohassan, 2013; Alharbi et al., 2019). Therefore, these different industries can be a source of these toxic elements and a reason for increasing the levels of these elements in the water samples of this site.

The high metal content in the water of Jeddah can be explained by the discharge of sewage water in this touristic area. Twenty one years ago, primary sewage-treated wastewater has been dumped into semi-enclosed coastal lagoon (2.5 km^2^) into the coastal water south of Jeddah (Southern Corniche). The sewage effluent rich in organic and metallic pollutants was retained within the lagoon (El Sayed, 2002; Basaham et al., 2009). Therefore, these sewage effluents can be a source of toxic elements in the water samples of Jeddah site. Also, the human activities in these areas such as transportation, construction, and manufacturing can produce large quantities of waste materials that cause water pollution (Bodin et al., 2013; Marchand et al., 2006; Alzahrani et al., 2018).

The higher metal content in the water samples of the upper part than the other parts might be due to its location close to the industrial and domestic pollution source, as also reported by Hamed and Emara (2006) in the northwestern part of Red Sea (Gulf of Suez).

## Conclusions

Contamination of mangrove forests in the Red Sea coast by potentially toxic elements can lead to adverse environmental, biological, economic, and social impacts on these ecosystems. Also, the high environmental stress in the arid zone mangrove lagoons due to high temperature, salinities, and high PTEs content can affect the plants, fish, and human health. Therefore, in our study we selected three mangrove forest lagoons (i.e., Al-Shuaiba, Yanbu, and Jeddah) in the Red Sea coast in Saudi Arabia and examined the levels of salinity, acidity, and the total content of Fe, Mn, Cu, Zn, Cd, Cr, Ni, and Pb in water. Based on our results, we can conclude that these lagoons, particularly Al-Shuaiba, suffer from highly salinity (69.4 dS/m). Also, these lagoons, particularly Yanbu and Jeddah contained high concentrations of total PTEs and the levels of these PTEs exceeded the standard limits of these two metals in the wastewater as reported by WHO and USEPA. The high concentrations of these metals in the water samples of the studied lagoons, particularly the upper part of Yanbu and Jeddah, may pose a great threat to these ecosystems. The mangrove ecosystems in the studied lagoons are highly affected by anthropogenic activities including urbanization, petrochemical industries, desalination and power generation plants, and wastewater treatment plants. For examples, the higher metal content in Yanbu site is mainly due to the petrochemical industries in this industrial area, while the high metal content in the water of Jeddah is likely due to the discharge of sewage water.

Our findings suggest that the high metal content in the water of these mangrove sites, particularly in Yanbu, should be considered due to the increase the potential environmental and human health risks in these ecosystems. These results may enable a more accurate prediction of water pollution in these mangrove forests in response to changing environmental, industrial, and social conditions. Moreover, this may help stakeholders and policy makers for creating new business opportunities for fish and wood producers and also for demonstrating sustainable and effective approaches for the management of these ecosystems.

More studies will be carried out to assess the content of these metals in sediment and different organs of mangrove plants and also to assess the pollution level in this ecosystem and the potentiality of mangrove plants for phytoremediation.
